# Supplementary material for: Intensifying rice production to reduce imports and land conversion in Africa
Source: Nat Commun. 2024 Jan 27;15:835. doi: 10.1038/s41467-024-44950-8 (PMC10821910; doi:10.1038/s41467-024-44950-8)
Supplement: Supplementary file 1 — Supplementary Information [file 41467_2024_44950_MOESM1_ESM.pdf]

Supporting Information for "Intensifying rice production to reduce imports and land conversion in Africa" by Yuan et al.

CONTENTS

1. **Rice production in the fifteen African countries …….........……….………. 2**
2. **Reference weather station and buffer zone selection……………..………. 2**
3. **Yield potential simulation ……………………..………………………..… 3**
4. **Weather data source……………...................………………………………... 4**
5. **Crop management and actual yield …………………………………..… 4**
6. **Sensitivity analysis ………………………………………………………..… 4**

Supplementary Figures 1-7…………………………………………....…..... 7-13

Supplementary Tables 1-7……………………………………………………… 14-21

Additional references associated with Supplementary Information 22

1. Rice production in the fifteen African countries

During the period of 2018 to 2020, Africa accounted for 10% and 5% of the global total rice harvested area and production, respectively[1]. Rice has become a staple food in Africa, and changes in consumption patterns and consumer preferences` have led to its increasing importance as a source of calories[2]. The fifteen countries included in our analysis (Egypt in North Africa, Burkina Faso, Côte d'Ivoire, Ghana, Mali, Niger, Nigeria, and Senegal in West Africa, and Ethiopia, Kenya, Madagascar, Rwanda, Tanzania, Uganda, and Zambia in East Africa) represent 65% and 80% of the total harvested rice area and production in Africa continent, respectively[1]. Average rice self-sufficiency ratio (estimated as the ratio between rice production and domestic consumption), harvested area, and annual production for each of the fifteen African countries are shown in Supplementary Table 1. The table also shows the percentage of harvested areas of irrigated and rainfed rice in relation to the total rice area in each country. The cropping systems in these countries are diverse, including different ecosystems (upland and lowland) and water regimes (rainfed and irrigated). For our assessment, we focused on the three types of rice production environments in Africa: irrigated, rainfed lowland, and rainfed upland. One or more environments were selected for each country, depending on their share of national rice area. Rice production in Africa involves both single- and double-season cropping, and Supplementary Fig. 1 shows the rice crop calendars for different regions in each country.

2. Reference weather station and buffer zone selection

Together with the expert knowledge from researchers at the Africa Rice Center (www.africarice.org) and national partners, a geospatial Spatial Production Allocation Model (SPAM 2010; www.mapspam.info) map was used to identify the major rice-producing regions, separately for irrigated and rainfed rice in each of the fifteen African countries (Supplementary Fig. 2). Reference weather station (RWS) buffers and climate zones (CZ) were selected by following previous studies[3,4]. In total, 45 RWS buffers were identified for irrigated rice, with one in Egypt and Kenya, two in Ghana and Senegal, three in Burkina Faso and Rwanda, four in Mali, Niger, and Tanzania, six in Nigeria, and 15 in Madagascar (Supplementary Fig. 2 and Table 2). A total of 45 RWS buffers were selected for rainfed lowland rice, including one in Ethiopia, two in Ghana and Zambia, three in Senegal, four in Burkina Faso, five in Mali and Tanzania, six in Uganda, eight in Nigeria, and nine in Côte

d'Ivoire. Similarly, a total of 26 RWS buffers were selected for rainfed upland rice, including one in Burkina Faso and Ethiopia, three in Mali, four in Tanzania, eight in Nigeria, and nine in Côte d'Ivoire. Overall, 45 RWS buffers were identified for irrigated rice, and 45 for rainfed rice. As a result, 35 and 36 CZ buffers were selected for irrigated and rainfed rice, respectively. The proportion of rice harvested areas located within selected buffers and CZs was 55% and 71% for irrigated rice and 28% and 54% for rainfed rice, respectively, across the 15 selected countries. Overall, the selected buffers and CZs accounted for 38% and 60% of the total rice production areas across the 15 countries, respectively.

3. Yield potential simulation

In the case of irrigated crops, we simulated yield potential, assuming no limitations by water deficit and/or excess and nutrients and no yield reductions due biotic factors such as weeds, insect pests, and pathogens[5,6]. Hence, for a given site each year, yield potential is determined by solar radiation, temperature, CO_2 concentration, and genetic traits influencing phenology and carbon assimilation and partitioning.

In the case of rainfed rice, we simulated the water-limited yield potential, which, besides accounting for all factors influencing yield potential, also considers the water supply and soil properties influencing the crop water balance such as texture and groundwater depth. For rainfed lowland rice, we simulated two different scenarios of groundwater depth throughout the entire rice growing season: shallow (40 cm) and deep (100 cm). The two scenarios represent rainfed favorable (shallow) and drought-prone (deep) environments. For the simulation of rainfed lowland rice, we assumed a non-puddled clayey loam soil with a bund height of 25 cm. We assumed that each groundwater scenario accounts for an equal proportion (50:50) of the rainfed lowland rice area in each buffer. In the case of rainfed upland rice, water-limited yield potential was simulated with a groundwater depth during the entire crop cycle of 1000 cm in a non-puddled sandy loam soil without a bund. Because upland rice fields are not puddled and tend to be located in coarse-textured soils with lowland rice fields, which are puddled and located in fine-textured soils, different percolation rates were used for rainfed lowland and upland rice: 4 and 240 mm day^{-1}, respectively[7].

We note that the yield potential of ten countries (Burkina Faso, Ghana, Mali, Niger, Nigeria, Ethiopia, Kenya, Tanzania, Uganda, and Zambia) was simulated using the latest daily weather data from 2000 to 2019. This simulation revealed that 75% of country-water regime

combinations exhibited no significant trend in yield potential. However, the remaining five countries (Egypt, Côte d'Ivoire, Senegal, Madagascar, and Rwanda) relied on previous simulation results based on weather data from 1995 and 2005. Even though the yield potential for the five countries was based on older, we found that this would not impact the findings of this study as we did not detect statistically significant trends in yield potential over time for the 10 countries for which we have 2000-2019 weather data. Hence, all 15 countries were included in our analysis.

Yield potential of irrigated rice showed lower year-to-year variation than water-limited yield potential of rainfed rice. Inter-annual coefficient of variation and semi-deviation for irrigated and rainfed rice was 6% versus 26% and 0.046 versus 0.269, respectively (Supplementary Table 3). Similarly, water-limited yield potential of rainfed lowland rice exhibited lower year-to-year variation, with an inter-annual coefficient of variation and semi-deviation of 23% and 0.251, respectively, as compared with 36% and 0.424 for rainfed upland rice.

4. Weather data source

Long-term (10+ years) daily weather data were available for all buffers in Burkina Faso, Ghana, Mali, Niger, Nigeria, Ethiopia, Kenya, Madagascar, Tanzania, Uganda, and Zambia during the period from 2000 to 2019 and in Côte d'Ivoire, Egypt, Rwanda, and Senegal from 1995 to 2005. Measured daily weather data were available for 23% of total RWS. In the case of those stations in which long-term measured daily weather data records were unavailable, two approaches were used to reach an acceptable number of years. For those stations where measured data were available for more than three but less than 10 years (76% of total RWS), weather records for missing time periods were generated based on existing measured weather data by a weather propagation methodology[8]. Following this approach, maximum and minimum temperatures from NASA-POWER gridded database are corrected based on the measured short-term data, while NASA-POWER solar radiation and precipitation are used without any correction[9]. This approach has shown to deliver estimates of yield potential that are closer to simulations based on measured weather data, compared with simulation based on uncorrected NASA-POWER data. In the case of RWS where measured weather data were available for less than three years or did not exist or were not accessible (1% of total RWS), uncorrected gridded weather data from NASA-POWER gridded database were used directly[8,10].

5. Crop management and actual yield

Crop management practices for each buffer were retrieved through agronomists from AfricaRice and national agricultural research institutes and extension agents. Requested information included: dominant crop sequences, ecosystems (upland/lowland), water regime (rainfed/irrigated) and proportion of each of them to the total harvested area, crop establishment method (transplanted/direct seeded), average sowing/transplanting dates, and dominant cultivar name and maturity. Dates of establishment (either direct seeding or transplanting) shown in Supplementary Fig. 1 correspond to dominate establishment date of each cropping system in each region reported by local agronomists and extension agents. Data on average farmers' yields were collected from national statistics, previous publications and databases, and local agronomists since crop yield data are not collected on a regular systematic way in some countries[11] (Supplementary Table 6). Data on farmers' yields were adjusted to a standard moisture content of 140 g H_2O kg^{-1} rice grain.

6. Sensitivity analysis

A sensitivity analysis was conducted to understand the impact of reduced yield potential and cropland expansion on total rice production and self-sufficiency ratio at the regional level in the context of climate change. We explored how different combinations of yield potential decrease and cropland expansion reduction would influence total rice production and self-sufficiency ratio under difference scenarios of yield intensification, including business-as-usual, half closure of the current exploitable yield gap, and full closure of the current exploitable yield gap. For the sensitivity analysis, 5-10% reduction was assumed in yield potential and rice area expansion rate due to climate change as compared with the baseline of estimated yield potential in our study and current rice area expansion rate (0.4 M ha per year), respectively[12-15]. The findings of our sensitivity analysis revealed that a 5% reduction in yield potential, coupled with a 5% or 10% decrease in area expansion, resulted in a decline of total rice production or the self-sufficiency ratio by less than 10% in each scenario of yield intensification, averaging at 5% and 7%, respectively (Supplementary Fig. 7). Similarly, a 10% decrease in yield potential, combined with a 5% or 10% decrease in area expansion, led to a reduction of total rice production or the self-sufficiency ratio by less than 15% in each yield intensification scenario, with average decreases of 7% and 10%, respectively. Hence,

the uncertainty in total rice production or self-sufficiency ratio due to reduced yield potential and cropland expansion associated with climate change is expected to be relatively small, especially when considering the magnitude of the current rice yield gaps (Fig. 2), suggesting that our findings are robust and, again, further emphasizing the need to close the current yield gaps.

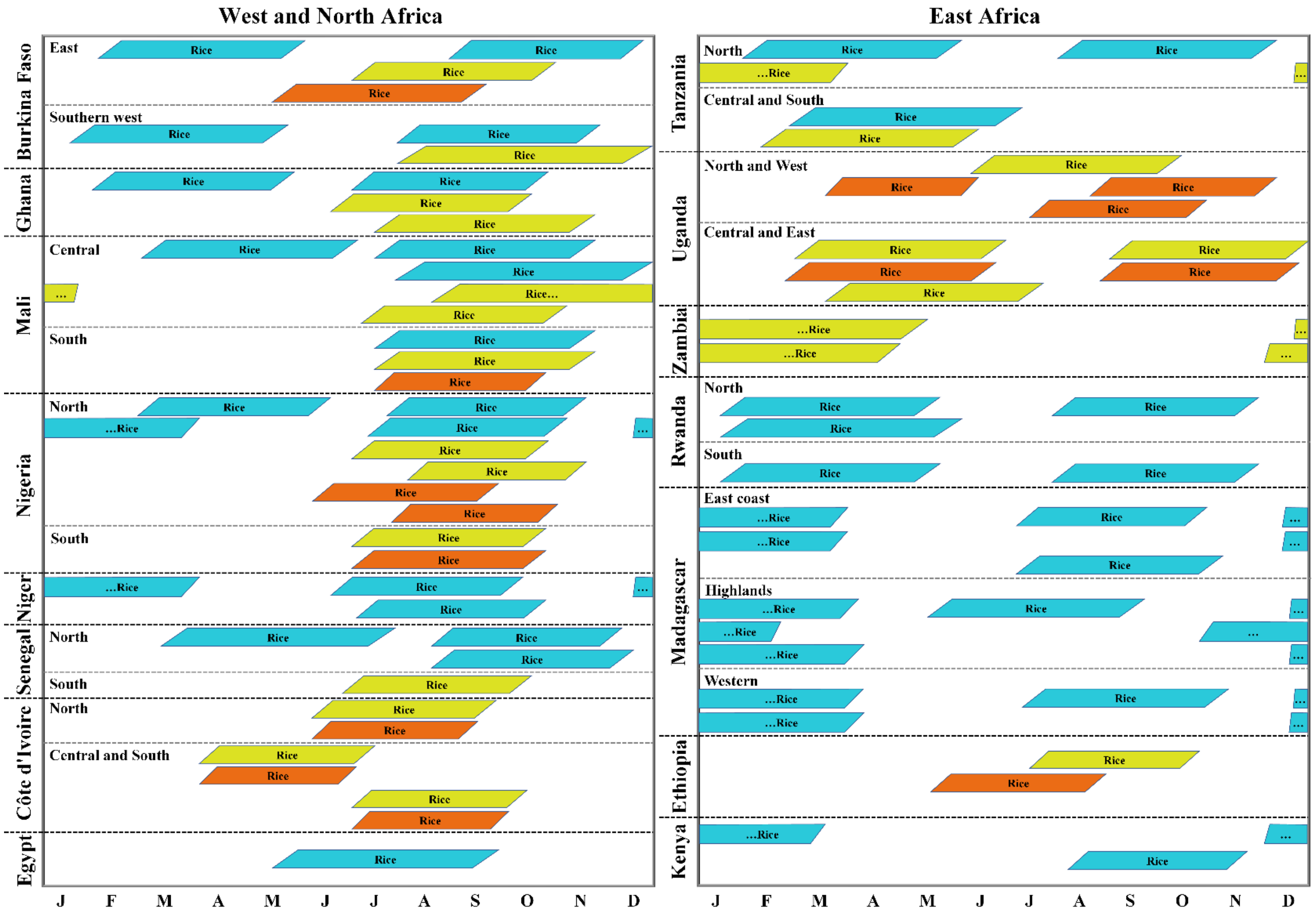

Supplementary Fig. 1 Dominant rice cropping systems in each country in West and North Africa (left panel) and East Africa (right panel). Boxes show approximate crop cycle length (from establishment to harvest maturity). Blue, yellow, and orange colors indicate irrigated, rainfed lowland, and rainfed upland rice, respectively.

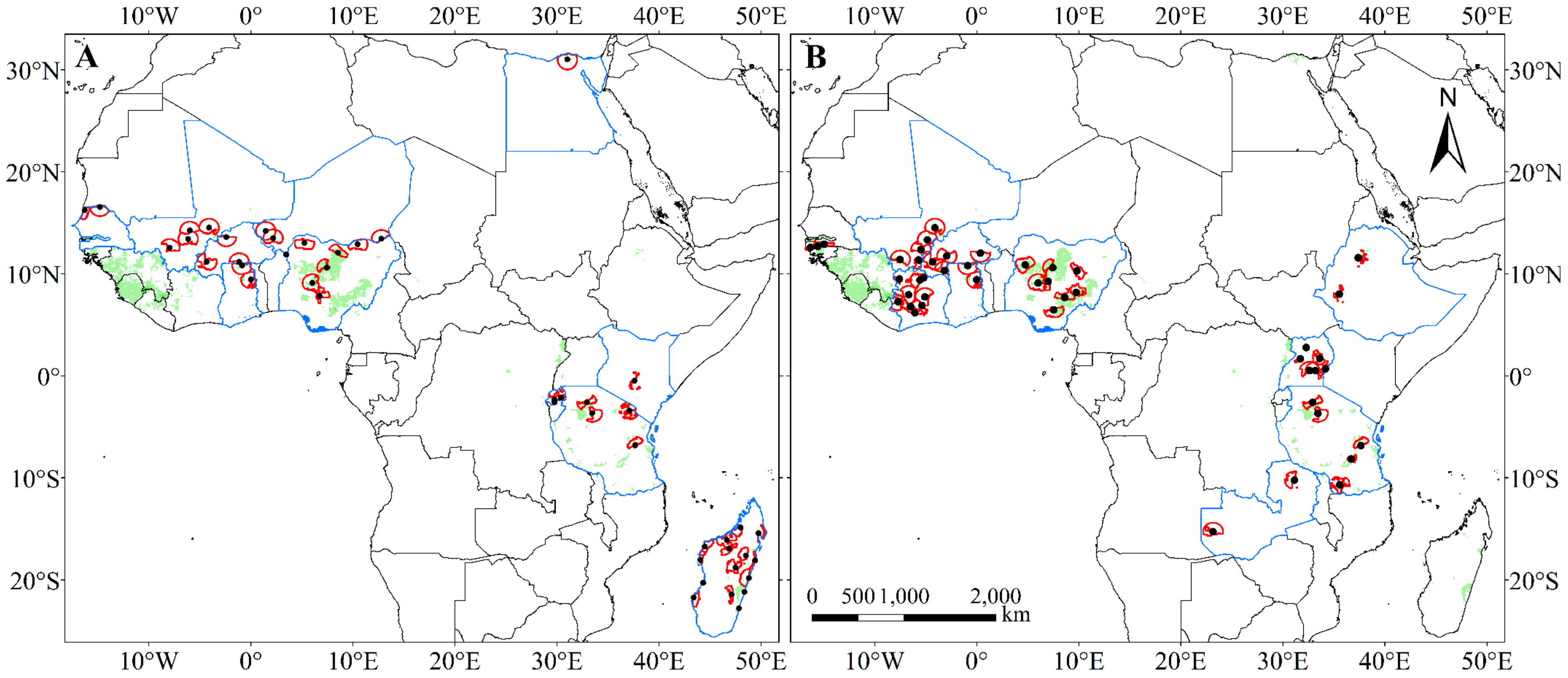

Supplementary Fig. 2 Selected buffers (red polygons) for (A) irrigated and (B) rainfed rice. Reference weather stations (black circles) and SPAM crop harvested area distribution (green) are shown (SPAM 2010; www.mapspam.info) in 15 selected rice-producing countries in Africa (blue polygons): Egypt in North Africa, Burkina Faso, Côte d'Ivoire, Ghana, Mali, Niger, Nigeria, and Senegal in West Africa, and Ethiopia, Kenya, Madagascar, Rwanda, Tanzania, Uganda, and Zambia in East Africa. The base map was applied without endorsement using data from the Database of Global Administrative Areas (https://gadm.org/).

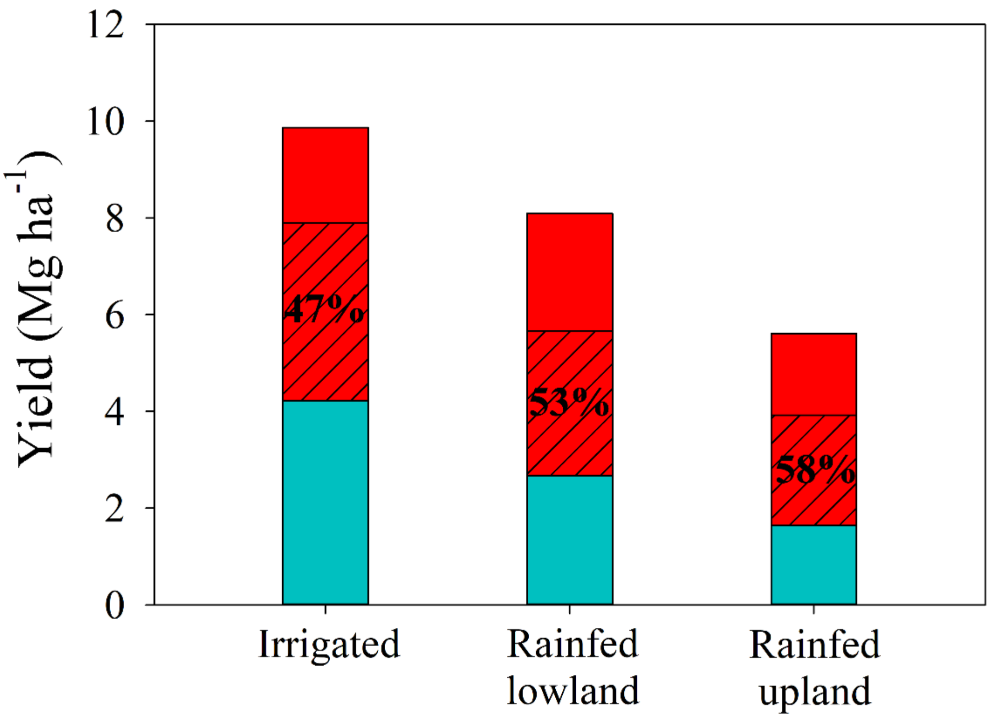

Supplementary Fig. 3 Average yield potential of irrigated and water-limited yield potential of rainfed lowland and upland rice in Africa. Blue and red portions of bars indicate the average farmers' yield and the yield gap. The hatched portion of the red bars represents the exploitable yield gap. Magnitude of exploitable yield gap, as percentage of the attainable yield, is also shown.

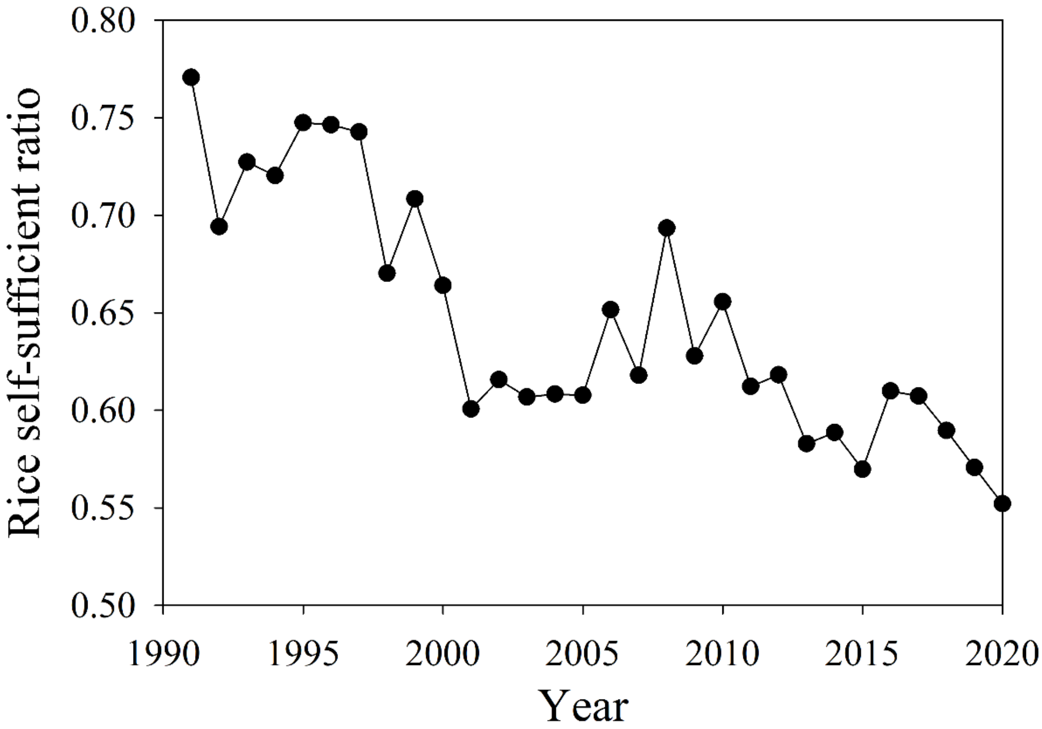

Supplementary Fig. 4 Trend in rice self-sufficiency ratio, which was estimated as the ratio of rice production to domestic rice consumption (calculated as rice production plus import minus export) for Africa during the past 30 years (1991-2020). Source: FAO[1] and USDA[16].

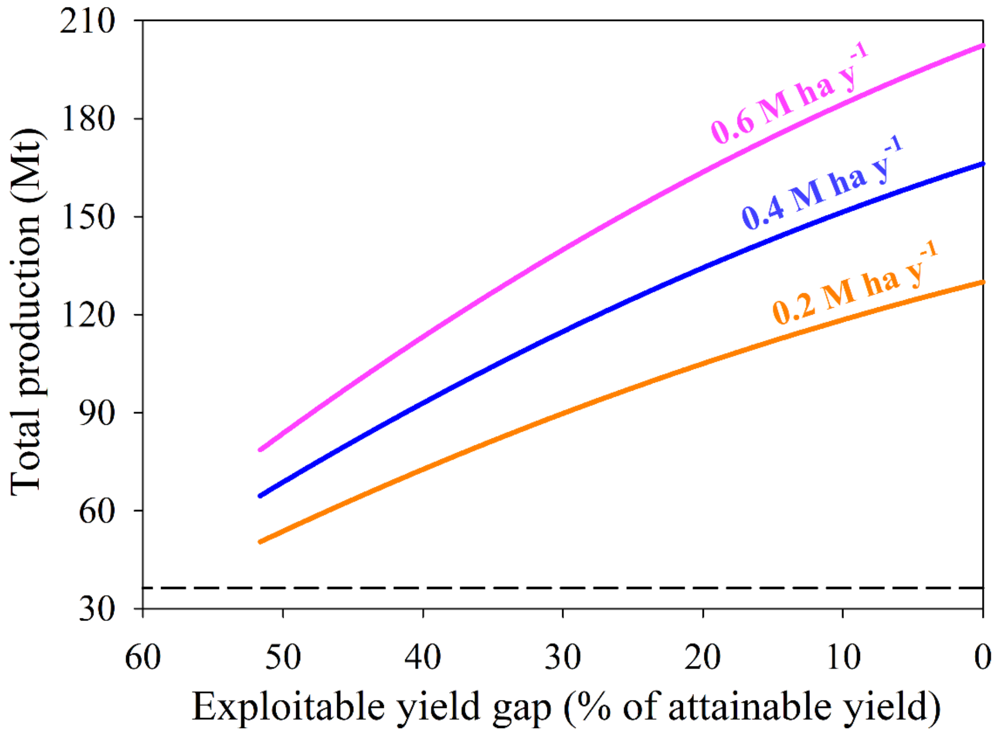

Supplementary Fig. 5 Assessment of total rice production by year 2050 under different scenarios of rice yield improvement and rice area expansion over the next 30 years. Dashed lines represent the current total rice production in Africa (average from year 2018-2020).

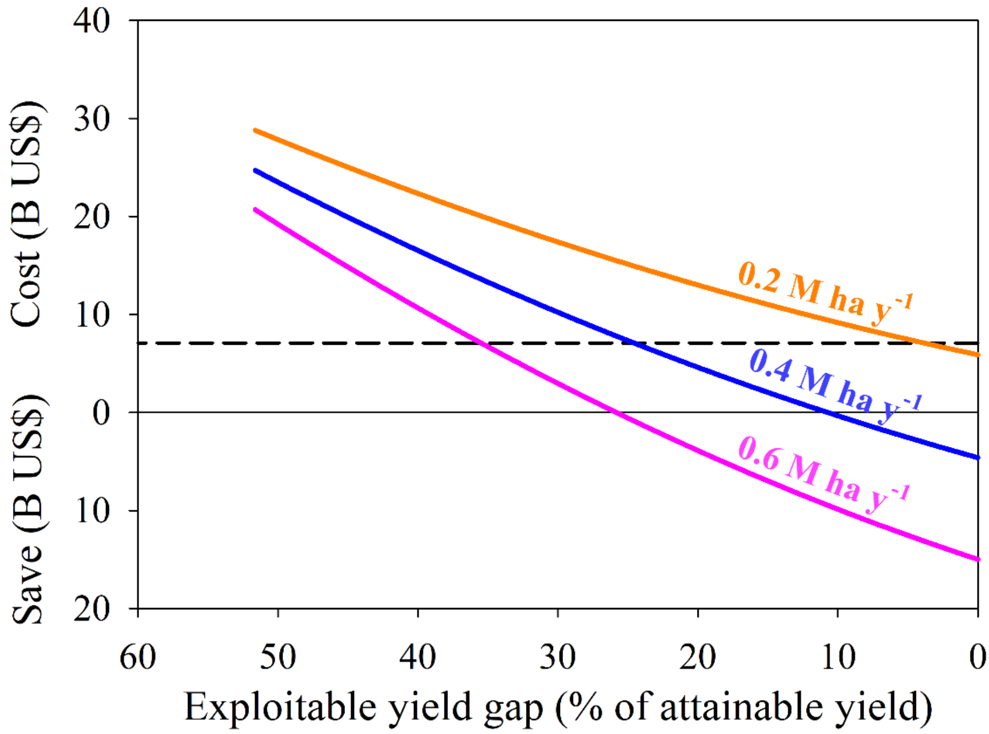

Supplementary Fig. 6 Economic costs associated with rice imports and exports by year 2050 for different scenarios of rice yield improvement and rice area expansion over the next 30 years. Dashed lines represent the current cost of rice imports (average from year 2018-2020).

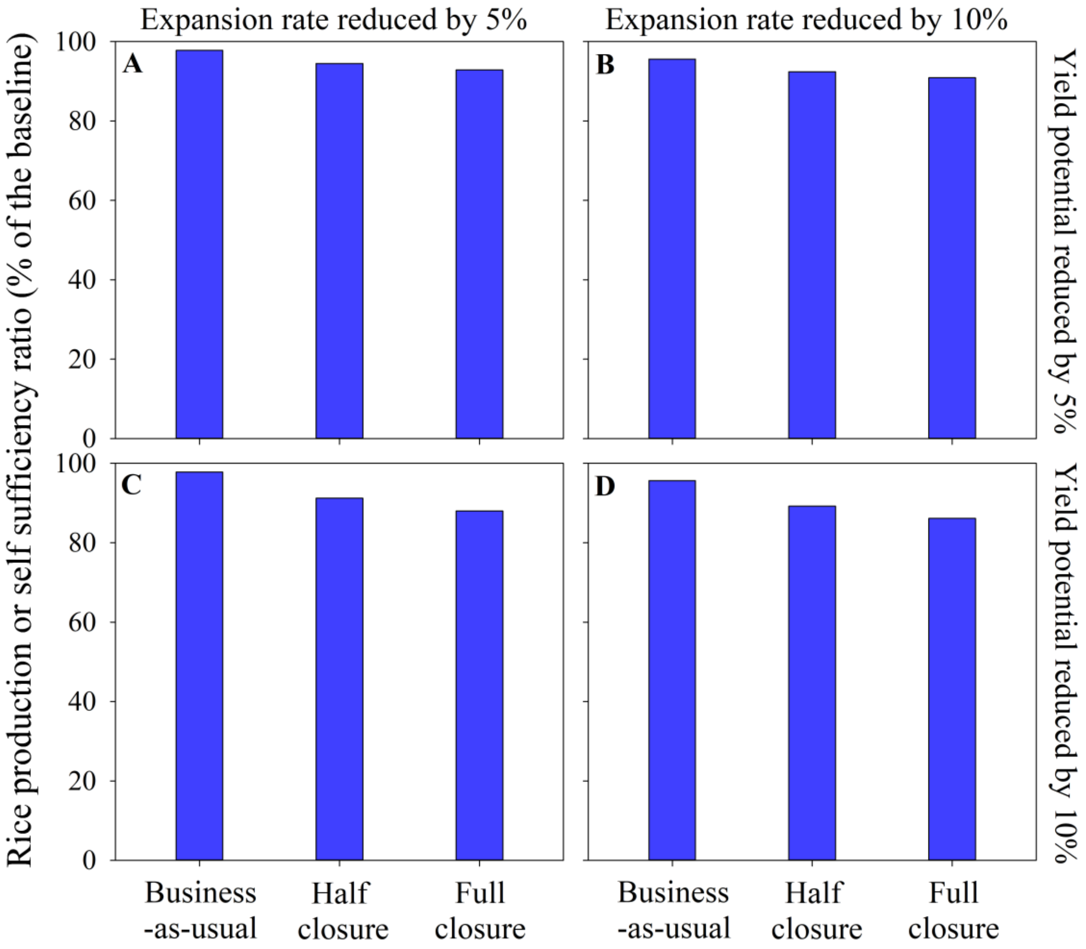

Supplementary Fig. 7 Relative change (%) in total rice production or self-sufficiency ratio at regional level due to 5-10% reduction in yield potential and rice area expansion rate due to climate change as compared with the baseline of estimated yield potential in our study and current rice area expansion rate (0.4 M ha per year) under different yield intensification scenarios (business-as-usual, half closure of the current exploitable yield gap, and full closure of the current exploitable yield gap): (A) both yield potential and area expansion rate are reduced by 5%, (B) yield potential and area expansion rate is reduced by 5% and 10%, respectively, (C) yield potential and area expansion rate is reduced by 10% and 5%, respectively, and (D) both yield potential and area expansion rate are reduced by 10%. For comparison purposes, the baseline corresponds to no changes in current yield potential and rice area expansion rate in each scenario.

Supplementary Table 1. Annual harvested rice area and total rice production during the 2018-2020 period and fraction of irrigated rice area to total rice in Burkina Faso, Côte d'Ivoire, Egypt, Ethiopia, Ghana, Kenya, Madagascar, Mali, Niger, Nigeria, Rwanda, Senegal, Tanzania, Uganda, and Zambia. Sources: FAO[1], USDA[16], Seck et al.[17], and van Oort and Zwart[18].

Country	Rice area (thousand ha)	Rice production (thousand tons)*	Irrigated rice area fraction (%)†
Burkina Faso	180	393	43
Côte d'Ivoire	647	1791	0
Egypt	470	4244	100
Ethiopia	61	177	0
Ghana	310	894	11
Kenya	26	151	100
Madagascar	1585	4056	100‡
Mali	931	3125	63
Niger	32	134	100§
Nigeria	4129	9155	10
Rwanda	32	121	100
Senegal	358	1237	46
Tanzania	1041	3309	30
Uganda	86	246	0
Zambia	27	36	0

* Rice production was reported in paddy rice at 140 g kg^{-1} seed moisture.

† Overall, we note that due to the small rainfed rice area, only irrigated rice was considered in Egypt, Kenya, Madagascar, Niger, and Rwanda. Similarly, only rainfed rice (lowland and/or upland) was considered in Côte d'Ivoire, Ethiopia, Uganda, and Zambia due to limited area of irrigated rice. Both irrigated and rainfed rice were considered in the remaining six countries: Burkina Faso, Ghana, Mali, Nigeria, Senegal, and Tanzania[17-19,20].

‡ We acknowledge that irrigated rice and rainfed lowland and upland rice are grown in Madagascar. However, since area of rainfed upland is limited and rainfed lowland rice often has access to water and are not water limited, separating irrigated and rainfed lowland rice becomes challenging[19]. As a result, we assumed rice in Madagascar is irrigated.

§ Although earlier research mentioned rainfed rice in Niger[19], we note that rice is primarily grown in flooded conditions. Thus, this study assumed that all rice area in Niger is irrigated.

Supplementary Table 2. The number of selected reference weather stations (RWS) for irrigated, rainfed lowland, and rainfed upland rice in the 15 selected rice-producing countries in Africa: Burkina Faso, Côte d'Ivoire, Egypt, Ethiopia, Ghana, Kenya, Madagascar, Mali, Niger, Nigeria, Rwanda, Senegal, Tanzania, Uganda, and Zambia.

Country	Irrigated	Rainfed lowland	Rainfed upland
Burkina Faso	3	4	1
Côte d'Ivoire	-	9	9
Egypt	1	-	-
Ethiopia	-	1	1
Ghana	2	2	-
Kenya	1	-	-
Madagascar	15	-	-
Mali	4	5	3
Niger	4	-	-
Nigeria	6	8	8
Rwanda	3	-	-
Senegal	2	3	-
Tanzania	4	5	-
Uganda	-	6	4
Zambia	-	2	-
**Total**	**45**	**45**	**26**

Supplementary Table 3. Temporal and spatial variation, quantified with the coefficient of variation (in %) and semi-deviation for yield potential of irrigated rice or water-limited yield potential of rainfed rice in the 15 selected rice-producing countries in Africa: Burkina Faso, Côte d'Ivoire, Egypt, Ethiopia, Ghana, Kenya, Madagascar, Mali, Niger, Nigeria, Rwanda, Senegal, Tanzania, Uganda, and Zambia and for the entire Africa.

Country	Irrigated			Rainfed		
	Temporal		Spatial	Temporal		Spatial
	CV	Semi-deviation [a]	CV	CV	Semi-deviation	CV
Burkina Faso	7	0.025	13	33	0.300	35
Côte d'Ivoire	-	-	-	11	0.106	26
Egypt*	1	0.016	-	-	-	-
Ethiopia	-	-	-	36	0.411	21
Ghana	4	0.045	12	20	0.129	23
Kenya*	11	0.089	-	-	-	-
Madagascar	3	0.025	18	-	-	-
Mali	9	0.080	13	35	0.301	25
Niger	5	0.054	4	-	-	-
Nigeria	5	0.036	7	15	0.112	27
Rwanda	0	0.014	11	-	-	-
Senegal	5	0.049	2	11	0.121	12
Tanzania	9	0.071	10	28	0.296	13
Uganda	-	-	-	31	0.350	18
Zambia	-	-	-	44	0.564	30
**Africa**	**6**	**0.046**	**8**	**26**	**0.269**	**22**

[a] Semi-deviation was calculated following a downside risk approach using the "PerformanceAnalytics" package in R software version 4.1.2[21].

Supplementary Table 4. Cross-validation of yield potential for irrigated rice or water-limited yield potential for rainfed rice (Mg ha^{-1}) as estimated in this study, based on measured yield (M) reported in other studies or simulated yield potential (S) from the same climate zone but in other regions of the world, for selected country-water regime combinations in Africa.

Country	Yield potential			Water-limited yield potential			Sources[†]
	Station/Climate zone[§]	This study	Other studies	Station/Climate zone	This study	Other studies	
Burkina Faso	Bobo-Dioulasso (I)	8.9	8.8 (M)	Gaoua (L)	6.4	6.0-7.0 (M)	Variety trial
Côte d'Ivoire	-	-	-	Man (U)	8.0	7.2 (M)	Saito et al.[22]
Egypt	Nile delta (I)	11.9	11.9 (M)	-	-	-	Yanni and Dazzo[23]
Ethiopia	-	-	-	Bahir Dar (L)	6.7	6-7.4 (M)	Variety trial, Saito et al.[22]
Ghana	10401 (I)	8.8	9.1 (S)	Yendi (L)	6.4	5.8 (M)	GYGA; Saito et al.[22]
Kenya	Embu (I)	9.8	9.2 (M)	-	-	-	Atera et al.[24]
Madagascar	9601/8901 (I)	9.5-11.1	9.3-12.4 (S)	-	-	-	GYGA
Mali	10201 (I)	10.5	10.5 (S)	10001 (L)	5.0	5.0 (S)	GYGA
Niger	Tillabery (I)	8.9	7.8 (M)	-	-	-	Tanaka et al.[25]
Nigeria	Kano (I)	8.4	8.6 (M)	Bauchi (L)	7.1	7.5 (M)	Variety trial; Saito et al.[22]
Rwanda	Kibungo (I)	9.4	8.4 (M)	-	-	-	Saito et al.[22]
Senegal	Fanaye (I)	7.4	6.4-7.8 (M)	Ziguinchor (L)	8.4	8.0 (M)	Saito et al.[26]; El-Namaky and Demont[27]; Faye et al.[28]
Tanzania	8301 (I)	11.6	9.8 (S)	9801 (L)	9.1	9.5 (S)	GYGA
Uganda	-	-	-	Namulonge (U)	4.2	4.4 (M)	Variety trial

[§] Code of climate zone is defined based on the Global Yield Gap Atlas Climate Zone (https://www.yieldgap.org/web/guest/climate-zones).

[†] Variety trial: Africa Rice Center variety trial database; GYGA: Global Yield Gap Atlas (https://www.yieldgap.org).

M: measured yields; S: simulated yields; I: irrigated rice: L: rainfed lowland rice; U: rainfed upland rice.

Supplementary Table 5. Baseline (2018-2020) and future (2050) population (million), per-capita rice demand (in kg paddy rice), and total annual national rice demand (in Mt paddy rice) at national level in the 15 selected rice-producing countries in Africa: Burkina Faso, Côte d'Ivoire, Egypt, Ethiopia, Ghana, Kenya, Madagascar, Mali, Niger, Nigeria, Rwanda, Senegal, Tanzania, Uganda, and Zambia and for the entire Africa.

Country	Population (million)*		Per-capita demand (kg)†		Total demand (Mt)‡	
	Baseline	Future	Baseline	Future	Baseline	Future
Burkina Faso	20	43	66	74	1.3	3.2
Côte d'Ivoire	26	51	144	174	3.8	8.9
Egypt	100	160	61	60	6.2	9.5
Ethiopia	112	205	12	12	1.2	2.4
Ghana	30	52	72	80	2.1	4.2
Kenya	53	92	20	37	1.1	3.4
Madagascar	27	54	179	236	4.8	12.7
Mali	20	44	179	212	3.6	9.2
Niger	23	66	29	35	0.7	2.3
Nigeria	201	401	54	63	11.0	25.4
Rwanda	13	23	19	39	0.2	0.9
Senegal	16	33	169	203	2.8	6.7
Tanzania	58	129	62	69	3.7	9.0
Uganda	44	89	7	9	0.3	0.8
Zambia	18	39	4	8	0.1	0.3
**Africa§**	**1328**	**2489**	**48**	**60**	**64**	**150**

* Current (2018-2020) and future (2050) population estimates are derived from the United Nations Population Dynamics[29] and the medium fertility variant of the United Nations Population Projections[30], respectively.† Current rice demand per capita was determined by dividing total domestic rice consumption based on USDA[11] by the current population[29]. Future per-capita rice demand was evaluated using a baseline of present per-capita rice demand and the relative change in per-capita rice demand projected by the IMPACT model (SSP2 scenario)[31]. The current and future per-capita rice demand were expressed in paddy rice by dividing originally reported milled rice from the USDA and the IMPACT by the rice milling rate[16,31], which was 0.65 in Burkina Faso, 0.65 in Côte d'Ivoire, 0.69 in Egypt, 0.65 in Ethiopia, 0.69 in Ghana, 0.66 in Kenya, 0.64 in Madagascar, 0.65 in Mali, 0.65 in Niger, 0.63 in Nigeria, 0.65 in Rwanda, 0.68 in Senegal, 0.66 in Tanzania, 0.65 in Uganda, and 0.66 in Zambia[16]. Average milling rate of 0.67 was used for other countries in Africa. ‡ The current national total rice demand was estimated based on the average annual national rice production, imports, exports, and stock change throughout the period of 2018-2020. Future national total rice demand by 2050 was calculated as the product of future population and future rice demand per capita by 2050.§ Current total rice demand in Africa was estimated as the average of annual regional rice production, import, export, and stock variation during 2018-2020. The projected total rice demand from the 37 countries included in USDA and IMPACT databases was multiplied by 1.05 to estimate the future total rice demand for the entire Africa by 2050, see *Methods* for details.

Supplementary Table 6. Time span and data sources of farmers' yield data for the 15 selected rice-producing countries in Africa: Burkina Faso, Côte d'Ivoire, Egypt, Ethiopia, Ghana, Kenya, Madagascar, Mali, Niger, Nigeria, Rwanda, Senegal, Tanzania, Uganda, and Zambia.

Country	Time span	Data sources
Burkina Faso	2005-2021	AfricaRice, RiceAdvice; AfricaRice, Rice Statistics Database; Nakano et al.[32]
Côte d'Ivoire	2007-2012	AfricaRice, Rice Statistics; ONDR, Système d'Information sur la filière Riz (SIR)
Egypt	2008-2012	United States Department of Agriculture (USDA)
Ethiopia	2007-2008	Agricultural Data Collection and Analysis (ADCA)
Ghana	2007-2012	AfricaRice, Rice Statistics Database; Abdul-Ganiyu et al.[33]; Dossou-Yovo et al.[34]
Kenya	2005-2009	Kenya National Bureau of Statistics (KNBS)
Madagascar	2004-2009	AfricaRice, Rice Statistics; Ministry of Agriculture, Livestock and Fisheries (MAEP), Recensement rizicole 2004/2005
Mali	2007-2021	AfricaRice, RiceAdvice; AfricaRice, Rice Statistics Database; Dossou-Yovo et al.[34]
Niger	2002-2008	National Office for Hydro-agricultural Developments of Niger (ONAHA); Local agronomists
Nigeria	2006-2021	AfricaRice, RiceAdvice; AfricaRice, Rice Statistics Database; National Bureau of Statistics of Nigeria; AfricaRice field survey
Rwanda	2005-2014	AfricaRice, Rice Statistics; Tanaka et al.[25]; Local agronomists
Senegal	2007-2011	National Society for the Development and Exploitation of the land of the Senegal River Delta (SAED)
Tanzania	2007-2013	Statistics Unit of the Ministry of Agriculture Food Security and Cooperatives of Tanzania; Dossou-Yovo et al.[34]; Local agronomists
Uganda	2006-2009	AfricaRice, Rice Statistics Database
Zambia	2010-2012	Local agronomists

Supplementary Table 7. Average rice yield (2018-2020) and yield trend during the past 30 years in Burkina Faso, Côte d'Ivoire, Egypt, Ethiopia, Ghana, Kenya, Madagascar, Mali, Niger, Nigeria, Rwanda, Senegal, Tanzania, Uganda, and Zambia. Sources: FAO[1].

Country	Average yield (Mg ha^{-1})	Yield trend* (kg ha^{-1} y^{-1})
Burkina Faso	2.18	0
Côte d'Ivoire	2.77	62
Egypt	9.01	0
Ethiopia	2.90	53
Ghana	2.88	43
Kenya	3.92†	0
Madagascar	2.72‡	0
Mali	3.36	0
Niger	4.37‡	69
Nigeria	2.22	0
Rwanda	3.80	65
Senegal	3.47	0
Tanzania	2.78‡	41
Uganda	2.84	59
Zambia	1.34	0

* The yield trend was set to zero when there is no trend or a declining trend in rice yield.

† Rice yield in Kenya was reported as the average during the period from 2010 to 2018 in which yield was relatively stable, as the yield was reported to be 60% higher in the most recent two years (2019-2020) than previous yield.

‡ Rice yield in Madagascar and Niger were reported as the average from the past five years (2016-2020) due to large yield variation.

Additional references associated with Supplementary Information

1. Food and Agriculture Organization of the United Nations, Crops and livestock products. https://www.fao.org/faostat/en/#data/QCL. Accessed 30 Dec 2022.
2. Ayanlade, A. & Radeny, M. COVID-19 and food security in Sub-Saharan Africa: implications of lockdown during agricultural planting seasons. *npj Sci. Food* **4**, 1-6 (2020).
3. van Bussel, L. G. et al. From field to atlas: upscaling of location-specific yield gap estimates. *Field Crops Res.* **177,** 98-108 (2015).
4. van Wart, J. et al. Use of agro-climatic zones to upscale simulated crop yield potential. *Field Crops Res.* **143,** 44-55 (2013).
5. Bouman, B. A. M. et al. *ORYZA2000: Modeling Lowland Rice* (International Rice Research Institute, 2001).
6. Lobell, D. B., Cassman, K. G. & Field, C. B. Crop yield gaps: their importance, magnitudes, and causes. *Annu. Rev. Environ. Resour.* **34**, 179 (2009).
7. van Oort, P. A. et al. Assessment of rice self-sufficiency in 2025 in eight African countries. *Glob. Food Secur-AGR* **5**, 39-49 (2015).
8. van Wart, J., Grassini, P., Yang, H., Claessens, L., Jarvis, A. & Cassman, K. G. Creating long-term weather data from thin air for crop simulation modeling. *Agr. Forest Meteorol.* **209**, 49-58 (2015).
9. NASA, NASA-Agroclimatology methodology. https://power.larc.nasa.gov/data-access-viewer/. Accessed 10 Sep 2022.
10. Grassini, P. et al. How good is good enough? Data requirements for reliable crop yield simulations and yield-gap analysis. *Field Crops Res.* **177**, 49-63 (2015).
11. African Development Bank, *Country Assessment of Agricultural Statistical Systems in Africa. Measuring the Capacity of African Countries to Produce Timely, Reliable, and Sustainable Agricultural Statistics* (Statistical Capacity Building Division of the Statistics Department, African Development Bank, Abidjan, Côte d'Ivoire, 2014).
12. van Oort, P. A. & Zwart, S. J. Impacts of climate change on rice production in Africa and causes of simulated yield changes. *Global Change Biol.* **24**, 1029-1045 (2018).
13. Zhao, C. et al. Temperature increase reduces global yields of major crops in four independent estimates. *Proc. Natl Acad. Sci. USA* **114**, 9326-9331 (2017).
14. Lotsch, A. *Sensitivity of cropping patterns in Africa to transient climate change. World Bank Policy Research Working Paper* (World Bank, Washington DC, USA, 2007).
15. Schleussner, C. F. et al. Crop productivity changes in 1.5 ^{o}C and 2 ^{o}C worlds under

climate sensitivity uncertainty. *Environ. Res. Lett.* **13**, 064007 (2018).

16. US Department of Agriculture, Foreign Agricultural Service. https://apps.fas.usda.gov/psdonline/app/index.html#/app/advQuery. Accessed 30 Dec 2022.
17. Seck, P. A., Diagne, A., Mohanty, S. & Wopereis, M. Crops that feed the world 7: Rice. *Food Secur.* **4**, 7-24 (2012).
18. van Oort, P. A. & Zwart, S. J. Impacts of climate change on rice production in Africa and causes of simulated yield changes. *Global Change Biol.* **24**, 1029-1045 (2018).
19. Diagne, A., Amovin-Assagba, E., Futakuchi, K. & Wopereis, M. C. *Estimation of cultivated area, number of farming households and yield for major rice-growing environments in Africa. Realizing Africa's rice promise* (CABI, Wallingford, UK, 2013).
20. Saito, K. et al. *Towards a better understanding of biophysical determinants of yield gaps and the potential for expansion of the rice area in Africa. Realizing Africa's rice promise* (CABI, Wallingford, UK, 2013).
21. R Core Team. *R: A language and environment for statistical computing*. R version 4.1.2. (R Foundation for Statistical Computing, Vienna, Austria, 2021).
22. Saito, K. et al. Yield-limiting macronutrients for rice in sub-Saharan Africa. *Geoderma* **338**, 546-554 (2019).
23. Yanni, Y. G. & Dazzo, F. B. Enhancement of rice production using endophytic strains of *Rhizobium leguminosarum* bv. trifolii in extensive field inoculation trials within the Egypt Nile delta. *Plant Soil* **336**, 129-142 (2010).
24. Atera, E. A., Onyancha, F. N. & Majiwa, E. B. Production and marketing of rice in Kenya: Challenges and opportunities. *J. Dev. Agric. Econ.* **10**, 64-70 (2018).
25. Tanaka, A. et al. On-farm rice yield and its association with biophysical factors in sub-Saharan Africa. *Eur. J. Agron.* **85**, 1-11 (2017).
26. Saito, K., Diack, S., Dieng, I. & N'Diaye, M. K. On-farm testing of a nutrient management decision-support tool for rice in the Senegal River valley. *Comput. Electron. Agr.* **116**, 36-44 (2015).
27. El-Namaky, R. A. & Demont, M. *Hybrid rice in Africa: challenges and prospects. Realizing Africa's rice promise* (CABI, Wallingford, UK, 2013).
28. Faye, O. N. et al. Status of Rice Cultivation and Breeding in Senegal. *J. Korean Soc. Int. Agric.* **32**, 381-389 (2020).
29. United Nations, Population Dynamics. https://population.un.org/wup/DataQuery. Accessed 10 Sep 2022.

30. United Nations, *World Population Prospects* (Department of Economic and Social Affairs, Population Division, UN, New York, New York, USA, 2022).
31. Robinson, S. et al. *The International Model for Policy Analysis of Agricultural Commodities and Trade (IMPACT)-Model Description for Version 3, IFPRI Discussion Paper 1483* (International Food Policy Research Institute, Washington DC, USA, 2015).
32. Nakano, Y., Bamba, Y., Diagne, A., Otsuka, K. & Kajisa, K. *The possibility of a rice green revolution in large-scale irrigation schemes in sub-Saharan Africa. Policy Research Working Paper 5560* (World Bank, Washington DC, USA, 2011).
33. Abdul-Ganiyu, S., Amaanatu, M. K. & Korese, J. K. Water use efficiency and productivity for rice (*Oryza Sativa*) in the Bontanga irrigation scheme of northern region of Ghana. *Agr. Sci. Res. J.* **2**, 362-368 (2012).
34. Dossou-Yovo, E. R., Vandamme, E., Dieng, I., Johnson, J. M. & Saito, K. Decomposing rice yield gaps into efficiency, resource and technology yield gaps in sub-Saharan Africa. *Field Crops Res.* **258**, 107963 (2020).
